# YouTube Videos on Nutrition and Dental Caries: Content Analysis

**DOI:** 10.2196/40003

**Published:** 2023-08-10

**Authors:** Memphis Long, Laura E Forbes, Petros Papagerakis, Jessica R L Lieffers

**Affiliations:** 1 College of Pharmacy and Nutrition University of Saskatchewan Saskatoon, SK Canada; 2 Department of Family Relations & Applied Nutrition University of Guelph Guelph, ON Canada; 3 Faculty of Dental Medicine Laval University Quebec City, QC Canada

**Keywords:** dental caries, diet, nutrition, YouTube, internet, consumer health information

## Abstract

**Background:**

Dental caries is the most common health condition worldwide, and nutrition and dental caries have a strong interconnected relationship. Foods and eating behaviors can be both harmful (eg, sugar) and healthful (eg, meal spacing) for dental caries. YouTube is a popular source for the public to access information. To date, there is no information available on the nutrition and dental caries content of easily accessible YouTube videos.

**Objective:**

This study aimed to analyze the content of YouTube videos on nutrition and dental caries.

**Methods:**

In total, 6 YouTube searches were conducted using keywords related to nutrition and dental caries. The first 20 videos were selected from each search. Video content was scored (17 possible points; higher scores were associated with more topics covered) by 2 individuals based on the inclusion of information regarding various foods and eating behaviors that impact dental caries risk. For each video, information on video characteristics (ie, view count, length, number of likes, number of dislikes, and video age) was captured. Videos were divided into 2 groups by view rate (views/day); differences in scores and types of nutrition messages between groups were determined using nonparametric statistics.

**Results:**

In total, 42 videos were included. Most videos were posted by or featured oral health professionals (24/42, 57%). The mean score was 4.9 (SD 3.4) out of 17 points. Videos with >30 views/day (high view rate; 20/42, 48% videos) had a trend toward a lower score (mean 4.0, SD 3.7) than videos with ≤30 views/day (low view rate; 22/42, 52%; mean 5.8, SD 3.0; *P*=.06), but this result was not statistically significant. Sugar was the most consistently mentioned topic in the videos (31/42, 74%). No other topics were mentioned in more than 50% of videos. Low–view rate videos were more likely to mention messaging on acidic foods and beverages (*P*=.04), water (*P*=.09), and frequency of sugar intake (*P*=.047) than high–view rate videos.

**Conclusions:**

Overall, the analyzed videos had low scores for nutritional and dental caries content. This study provides insights into the messaging available on nutrition and dental caries for the public and guidance on how to make improvements in this area.

## Introduction

Dental caries (or tooth decay) occurs when dietary fermentable carbohydrates (eg, simple sugars) are metabolized by bacteria in the mouth (eg, *Streptococcus mutans*) to produce a highly acidic environment that can degrade tooth structures (eg, enamel) [1]. Dental caries is the most common disease worldwide. According to the 2019 Global Burden of Disease Study, 2.0 billion people worldwide had untreated dental caries in permanent teeth, and 0.5 billion children aged 0 to 14 years had untreated caries in their deciduous teeth [2]. Untreated dental caries is more common than cardiovascular diseases, diabetes, cancers, mental disorders, and chronic respiratory diseases worldwide [3]. The World Health Organization recommends that oral health care become part of universal health care and that there is an increased emphasis on the prevention of oral diseases [4].

Although dental caries can be attributed to numerous interrelated factors described elsewhere [1,5], many foods and dietary habits have been identified as important risk factors. Sugar, which is a fermentable carbohydrate, is a major driving force for the development of dental caries [6,7]. The World Health Organization strongly recommends that children and adults consume <10% of their calories as free sugars because of the association between this dietary component and dental caries. They also conditionally recommend reducing free sugar intake to <5% of the total energy consumed because of an additional protective effect of lower intakes on dental caries risk [8]. Sugary drinks (eg, soft drinks and juice) have also been linked to dental caries, and limiting the consumption of these drinks has been recommended [1,9-14]. Furthermore, foods that are both sugary and starchy (eg, cakes and donuts) are thought to be more cariogenic than foods containing sugar alone; this outcome is likely owing to the sugar being retained on teeth for longer periods due to the stickiness of the starch [15,16]. In addition, more frequent consumption of sugar (including consumption between meals) is associated with an increased risk of dental caries than less frequent consumption of sugar [1,17]. Poor-quality diets can also cause nutrient deficiencies (eg, vitamin D and calcium) that can cause issues with tooth formation and mineralization, making them susceptible to caries development [7,18].

Dietary factors can also prevent the development of dental caries. Foods that are thought to benefit teeth are whole grains, fruits, vegetables, high-quality proteins, and dairy products such as cheese and milk; spacing out meals is also thought to be beneficial [1,14,19,20]. Furthermore, xylitol is thought to be beneficial for dental caries prevention through different mechanisms, including replacing sugar intake in the diet; stimulation of saliva; and inhibition of the growth of cariogenic bacteria [21]. Although diet is an important determinant of dental caries, many studies have reported that health professionals experience substantial barriers in providing diet counseling for this issue, and often, this service may not be provided [22].

Previous research has found that it is common for the public to access web-based sources (eg, internet and social media) to obtain health information [23-25]. For example, Shahab et al [23] found that in the United States, 78.2% of individuals who had ever used the internet had used this source in the previous year to access health information. The Tracking Nutrition Trends survey conducted by the Canadian Foundation for Dietetic Research in 2015 found that 49% of survey respondents from Canada used the internet, social media, and blogs to obtain information on food and nutrition. They also found that this activity was more common among the younger respondents [26]. There are numerous reasons why members of the public may seek health information from web-based sources, including to obtain more knowledge on a health condition, supplement information obtained from health providers, explore embarrassing topics, and obtain support from others [27,28]. However, despite the popularity of web-based information, there are concerns with accessing these sources, including the presence of misinformation and potential harms of making decisions based on unsubstantiated claims [27,29].

YouTube is one source of web-based information that deserves attention. It is a video sharing platform founded in 2005 and is the second most highly trafficked website globally, with 34.6 billion visits each month [30]. In 2020, there were 2.3 billion users of YouTube globally, and this has steadily increased over the last several years [31]. The content uploaded to YouTube is extensive. For example, for every minute as of February 2020, a total of 500 hours of video content was uploaded [32]. There are many reasons why people use YouTube, including to learn new things, problem-solving, entertainment, self-care (eg, destress and relaxation), and to improve skills [33]. In a 2019 report, approximately 70% of YouTube users reported that this platform is the first website they go to when trying to learn [34]. YouTube can also be easily accessed through different devices, including computers, tablets, and mobile phones. Substantial interest has been generated around the use of YouTube for health-related purposes. To date, a few studies have shown that YouTube videos can be beneficial for improving health-related knowledge, attitudes, and behaviors [28]. However, despite the popularity of this platform and the interest in its use for health-related purposes, the content of YouTube videos is not reviewed to ensure accuracy and comprehensiveness.

To date, several studies have been conducted on the content of health information available on YouTube. These studies have been summarized in different review articles [28,35-38]. These articles have reported that, in general, videos do not comprehensively cover various health topics and that the content quality of videos varies widely, with many studies reporting a high prevalence of poor-quality videos or nonuseful videos and a low prevalence of good-quality videos. However, some high-quality videos are available in some topic areas [37,38]. In addition, many studies have found either no relationship between video quality and engagement (eg, views and likes) or a negative relationship (ie, as quality decreases, engagement increases) [38]. These articles have also found that videos tend to be of higher quality when they feature health professionals (eg, physicians) or health organizations [37]. Although numerous studies have assessed the content of various types of health-related YouTube videos, to our knowledge, no studies have examined the content of YouTube videos related to nutrition and dental caries. Owing to the high prevalence of dental caries worldwide, the strong relationship that diet has with dental caries, the popularity of YouTube, and the barriers experienced by health professionals providing support on this issue, information on this topic is needed.

The purpose of this study was to analyze the content of YouTube videos regarding dental caries and nutrition that are easily accessible using default search settings. We were also interested in examining nutrition messaging according to creator type and engagement.

## Methods

### Ethical Considerations

This study was exempt from ethical review from the University of Saskatchewan Behavioural Ethics Office as per Article 2.2 of the Tri-Council Policy Statement (TCPS): Ethical Conduct for Research Involving Humans—TCPS 2 (2018) [39].

### Video Selection

Our strategy was to search for YouTube videos that would be most accessible to the public searching for educational content regarding nutrition and dental caries. Google Keyword Planner [40] was used to select 2 dental caries–related keywords and 3 nutrition-related keywords. For dental caries, the 2 top keywords associated with this concern were *tooth decay* and *dental cavities*. The top 3 keywords for nutrition were *nutrition*, *diet*, and *food*. This resulted in a total of 6 searches: *tooth decay and nutrition*, *tooth decay and diet*, *tooth decay and food*, *dental cavities and nutrition*, *dental cavities and diet*, and *dental cavities and food*. Videos were eligible for inclusion if they were in English, <20 minutes in duration, and included information about nutrition and dental caries. We chose <20 minutes in duration as the inclusion criteria because similar time frames have been used in previous related work [41,42]. A 2018 study also found that 90% of respondents preferred instructional and informational videos to be <20 minutes [43].

The YouTube searches were conducted on May 17, 2021, using the default settings on YouTube to best replicate the search strategy used by the public. The searches were conducted by ML, a female undergraduate nutrition student, using Google Chrome’s Incognito mode to prevent bias when conducting the searches. ML opened a new incognito window to complete each search. Each of the 6 YouTube searches were completed in a sequence, and the first 20 videos were recorded from each search. The first 20 videos were chosen because similar numbers have been used in other related studies [44-46]. We also chose the first 20 videos because previous work has found that most people who use the internet do not look past the first search results page [47]. For each video, the title, publisher, country of origin, total number of views, date posted, URL, length in minutes, whether the video was an animation, and the number of likes and dislikes were recorded by ML in Microsoft Excel 365. Transcripts for each video were also downloaded from the YouTube website.

### Video Scoring System

Owing to the numerous dietary factors that can affect the risk of dental caries, a scoring system was developed to be used for this study. This type of approach (scoring system or presence or absence of content in videos) has been used in other related YouTube content analysis studies [48-52]. The scoring system had 17 possible points, with higher scores indicating that more topic areas were covered. Table 1 lists each of the topic areas. The inclusion of misinformation in videos was not considered in the scoring tool. This approach has also been used elsewhere [51].

**Table 1:** Scoring system to assess messaging in the included YouTube videos (total possible score: 17 points).

Message assessed in each video		Score, n
Dental caries mechanism		1
**Factors that increase the risk of dental caries (or poor oral health)**		
	Acidic foods and beverages	1
	Any mention of sugar	1
	Sugary drinks (eg, soda, fruit juices, energy drinks, and sweetened coffee and sweetened tea)	1
	Sticky foods (eg, dried fruit)	1
	Frequency of sugar intake (eg, frequent and prolonged intake of simple sugars or limiting snacking or eating sugary foods with meals or eating sticky foods alone)	1
	Candy (either in general or specific types of candy)	1
	Snack foods high in sugar and starch (eg, cookies, cakes, and pastries)	1
**Factors that reduce the risk of dental caries (or promote good oral health)**		
	Chewing sugar-free gum or eating sugar-free candy or xylitol	1
	Vegetables and fruit (including specific vegetables and fruits)	1
	Protein from high-quality sources (eg, meats, nuts, seeds, and legumes)	1
	Whole grains	1
	Water	1
	Dairy products (both in general or mentioning specific products)	1
	Drink beverages with a straw	1
	Brush teeth after meals or brush teeth at least 2 times/day	1
	Mention food guide or food label reading	1

This scoring system was developed partially based on the Academy of Nutrition and Dietetics’ most recently published position paper on Oral Health and Nutrition [1], which identifies dietary patterns and eating behaviors associated with an increased and decreased risk of dental caries. In this position paper, dietary patterns and behaviors that were identified as causing an increased risk of dental caries included sugar intake, sugary beverage intake, candy intake, starchy and sugary food intake, sticky food intake, and frequency of consuming sugary foods and beverages. For dietary patterns and behaviors associated with decreased risk, the position paper included sugar-free gum and candies, vegetables and fruit, high-quality protein foods, and whole-grain foods.

Dietary factors were also added to the scoring tool based on content from other high-quality evidence-based sources related to nutrition and dental caries, including the National Health Service Health Scotland Oral Health and Nutrition Guidance for Professionals June 2012 [14], the 2015 Joint Position Statement on Oral Health and Nutrition from the Dietitians Association of Australia and Dental Health Services Victoria [20], and a Chairside Dietary Assessment tool developed by a dietitian published by the *Journal of the American Dental Association* [53]. These additional protective factors included dairy products, water, and drinking with a straw. Acidic foods and beverages were also included because they have been found to cause dental erosion [54,55]. Caution surrounding acidic foods and beverages is mentioned by both the Scottish and Australian guidelines listed earlier [14,20]. We also examined videos for mention of the food guide or food label reading, as these are common recommendations for general healthy eating and were mentioned in the Scottish guidelines [14], and for information about toothbrushing [14,20]. The recommendation to drink with a straw was found in the chairside assessment tool [53]. In addition, the mechanism of dental caries was also included in the scoring system (ie, including information about how bacteria in the mouth convert sugar into acid and damage tooth structures).

### Data Analysis

Videos were scored using the 17-point scoring system independently by 2 individuals (ML and JRLL) using information presented in either text listed in the video or what was said verbally. Discrepancies were discussed until a consensus was reached.

Information on video characteristics (ie, view count, length, number of likes, number of dislikes, video age, viewing rate [views/day; calculated by taking the number of views and dividing by number of days since the video was uploaded] [48], like rate [likes/view; calculated by taking the number of likes and dividing by the number of views], and dislike rate [dislikes/view; calculated by taking the number of dislikes and dividing by the number of views]) were summarized using descriptive statistics (mean, SD, median, and range) determined using Microsoft Excel 365 and SPSS (version 28; IBM Corp).

Each video was categorized into 1 of 4 groups based on the author or presenter featured in the video. The four groups were as follows: (1) oral health professionals (OHPs; eg, dentists, dental hygienists, dental practice groups, dental offices, or commercial content reviewed by OHPs), (2) health professionals who are not OHPs including complementary and alternative medicine providers (eg, microbiologists, chiropractors, and naturopaths), (3) government (videos posted by government sources that could feature any type of health professional), and (4) no health professional credentials or unknown credentials (eg, social media influencers with no credentials and bloggers). Videos were categorized into 2 roughly equal-sized groups based on view rate to examine differences between the most viewed videos compared with less commonly viewed videos.

Inferential statistics were determined using SPSS Statistics (version 28). Fisher exact test was used to determine whether there were significant differences between categorical variables, and the Mann-Whitney *U* test and Kruskal-Wallis test were used to determine whether there were significant differences between continuous variables. The Bonferroni correction was used to correct for multiple comparisons. Spearman correlations were used to examine the relationships between 2 continuous variables. *P* values of <.05 were considered significant.

## Results

### Search Results

In total, 120 videos from the 6 searches were considered for inclusion; 78 (65%) videos were removed from the analysis because they (1) were duplicate videos (n=65, 54.2%) or (2) did not meet inclusion criteria (n=13, 10.8%; ie, video did not mention anything related to diet and dental caries, n=9, 7.5%; video was >20 min, n=3, 2.5%; and video was not in English, n=1, 0.8%). After these videos were removed, 42 videos were eligible for analysis.

### Video Characteristics

Characteristics of the included videos are provided in Table 2. Most videos were posted by or featured OHPs (24/42, 57%), followed by those with no health professional credentials or unknown credentials (10/42, 24%), health professionals who were not OHPs including complementary and alternative medicine providers (6/42, 14%), and the government (2/42, 5%). Notably, 17% (7/42) of the videos were presented as cartoons.

Most videos originated from the United States (25/42, 60%), followed by the United Kingdom (4/42, 10%), India (4/42, 10%), Canada (3/42, 7%), Australia (2/42, 5%), Indonesia (1/42, 2%), and Italy (1/42, 2%). For 5% (2/42) of the videos, we were unable to identify the country of origin. Included videos were on average 4 minutes and 40 seconds in length (SD 3 min and 9 s; range 47 s to 16 min and 35 s) and had been posted for a median of 926.5 (range 164-3917) days.

**Table 2:** Characteristics of the YouTube videos on nutrition and dental caries included for the analysis (n=42).

			All videos (n=42)		OHPs^a^ (n=24)		Health professionals who are not OHPs including complementary and alternative medicine providers (n=6)		Government (n=2)		No health professional credentials or unknown credentials (n=10)
**Video age (days)**											
	Median (range)	926.5 (164-3917)		927 (164-3917)		1694 (663-2160)		580 (356-804)		778 (192-3255)	
	Mean (SD)	1202 (952)		1257 (1055)		1609 (569)		580 (317)		949 (906)	
**Length**											
	Median (range)	3 min and 56 s (47 s to 16 min and 35 s)		3 min and 45 s (1 min and 2 s to 16 min and 35 s)		4 min and 54 s (2 min and 29 s to 9 min and 14 s)		3 min and 4 s (1 min and 51 s to 4 min and 16 s)		3 min and 41 s (47 s to 9 min and 18 s)	
	Mean (SD)	4 min and 40 s (3 min and 9 s)		4 min and 58 s (3 min and 36 s)		5 min and 8 s (2 min and 26 s)		3 min and 4 s (1 min and 43 s)		4 min and 0 s (2 min and 37 s)	
**View count**											
	Median (range)	21,533 (394-3,768,733)		17,741 (394-1,512,464)		718,780 (3080-3,768,733)		11,526 (4564-18,488)		52,383 (1485-1,854,382)	
	Mean (SD)	292,689 (706,004)		119,821 (320,627)		1,150,249 (1,403,640)		11,526 (9846)		249,270 (569,009)	
**Viewing rate (views/number of days since posting)**											
	Median (range)	29.6 (0.3-4863.2)		15.6 (0.3-4863.2)		490.9 (1.4-2252.7)		28.8 (5.7-51.9)		44.8 (6.0-2499.2)	
	Mean (SD)	375.1 (945.6)		343.8 (1085.5)		687.2 (813.1)		28.8 (32.7)		321.5 (773.2)	
**Number of likes**											
	Median (range)	422 (0-33,000)		257 (0-8100)		20,500 (43-30,000)		12 (0-24)		609 (28-33,000)	
	Mean (SD)	4130 (8832)		1145 (2194)		17,491 (12,905)		12 (17.0)		4100 (10,196)	
**Like rate**											
	Median (range)	0.015 (0-0.047)		0.015 (0-0.034)		0.020 (0.0080-0.041)		0.0026 (0-0.0053)		0.018 (0.005-0.047)	
	Mean (SD)	0.017 (0.012)		0.016 (0.010)		0.024 (0.014)		0.0026 (0.0037)		0.020 (0.013)	
**Number of dislikes**											
	Median (range)	14 (0-1200)		12 (0-633)		429 (0-1200)		0 (0-0)		18 (0-1100)	
	Mean (SD)	159 (325)		80 (180)		553 (532)		0 (0)		146 (339)	
**Dislike rate**											
	Median (range)	0.00057 (0-0.0046)		0.00061 (0-0.0046)		0.00060 (0-0.00075)		0 (0-0)		0.00055 (0-0.0012)	
	Mean (SD)	0.00063 (0.00074)		0.00075 (0.00092)		0.00049 (0.00028)		0 (0)		0.00054 (0.00038)	

^a^OHP: oral health professional.

Overall, the 42 included videos had 12,292,954 total views recorded. Videos published by health professionals who are not OHPs (including complementary and alternative medicine providers) had the most views (median 718,780, range 3080-3,768,733 views/video; total views: 6,901,491), followed by videos published by or featuring those with no health professional credentials or unknown credentials (median 52,383, range 1485-1,854,382; total views: 2,492,704), videos that were published by or featured OHPs (median 17,741, range 394-1,512,464; total views: 2,875,707), and videos from the government (median 11,526, range 4564-18,488; total views: 23,052). The mean viewing rate (views/day) was similar between videos posted by those with no health professional credentials or unknown credentials (321.5, SD 773.2; range 6.0-2499.2) and OHPs (343.8, SD 1085.5; range 0.3-4863.2); however, videos by health professionals who are not OHPs (including complementary and alternative medicine providers) had a higher mean view rate (mean 687.2, SD 813.1; range 1.4-2252.7).

### Nutrition Messaging

The mean video score for all included videos (42/42, 100%) was 4.9 (SD 3.4; of a maximum possible total of 17), with scores varying from 0 to 13. Table 3 provides a breakdown of the information on scoring by creator type. Videos published by the government and OHPs had a higher mean score (government: 6.5, SD 0.7; OHPs: 5.7, SD 3.8) compared with the scores of videos published by other health professionals (including complementary and alternative medicine providers) or those with no health professional credentials or unknown credentials (other health professionals: 4.0, SD 1.3; no health professional credentials or unknown credentials: 3.4, SD 3.3). However, there was no statistically significant difference in the video scores between the creator type (*P*=.29). Of note, 14% (6/42) of the videos had a score of 0; these videos were published by individuals with no health professional credentials or unknown credentials (n=3, 50%) and OHPs (n=3, 50%).

We investigated the correlation between total video scores and public engagement with videos. No significant Spearman correlations were found between the total video score and total views (−0.114; *P*=.47), view rate (−0.196; *P*=.21), total likes (−0.200; *P*=.20), like rate (−0.202; *P*=.20), total dislikes (−0.156; *P*=.32), and dislike rate (−0.199; *P*=.21).

To further examine nutrition messaging and video engagement, we divided all videos (42/42, 100%) into 2 similar-sized groups based on view rate. The high–view rate category (>30 views/day; 20/42, 48% videos) consisted of 7 videos by OHPs, 7 videos from the no health professional credentials or unknown credentials category, 5 videos by health professionals who are not OHPs (including complementary and alternative medicine providers), and 1 video by the government. The low–view rate category (≤30 views/day; 22/42, 52% videos) consisted of 17 videos by OHPs, 3 videos in the no health professional credentials or unknown credentials category, 1 video by health professionals who are not OHPs (including complementary and alternative medicine providers), and 1 video by the government. Videos with >30 views/day (20/42, 48% videos) had a mean score of 4.0 (SD 3.7) compared with videos with ≤30 views/day (22/42, 52%), which had a mean score of 5.8 (SD 3.0); there was a trend toward the scores being different between groups (*P*=.06; Table 4), but this result was not statistically significant.

Table 5 provides an in-depth breakdown of the different nutrition messages for all videos (42/42, 100% videos). In addition, information on the breakdown of messaging in low–view rate videos (≤30 views/day) versus high–view rate videos (>30 views/day) is also presented.

**Table 3:** Scores of YouTube videos on nutrition and dental caries by type of creator (n=42).

		All videos (n=42)	OHPs^a^ (n=24)	Health professionals who are not OHPs including complementary and alternative medicine providers (n=6)	Government (n=2)	No health professional credentials or unknown credentials (n=10)
**Total score (out of 17)**						
	Median (range)	4.5 (0-13)	5.5 (0-13)	3.5 (3-6)	6.5 (6-7)	3.5 (0-10)
	Mean (SD)	4.9 (3.4)	5.7 (3.8)	4.0 (1.3)	6.5 (0.7)	3.4 (3.3)

^a^OHP: oral health professional.

**Table 4:** Scores of YouTube videos on nutrition and dental caries by view rate (n=42).

		All videos	Low video view rate: ≤30 views/day (range 0.3-29.8; n=22)	High video view rate: >30 views/day (range 35.5-4863.2; n=20)
**Total score (out of 17)^a^**				
	Median (range)	4.5 (0-13)	6 (0-11)	3.5 (0-13)
	Mean (SD)	4.9 (3.4)	5.8 (3.0)	4.0 (3.7)

^a^*P*=.06 for the difference between high–view rate videos and low–view rate videos (Mann-Whitney *U* test).

**Table 5:** Nutrition and dental caries messaging included in the analyzed YouTube videos by view rate (n=42).

		All videos (n=42), n (%)	Low video view rate: ≤30 views/day (range 0.3-29.8; n=22), n (%)	High video view rate: >30 views/day (range 35.5-4863.2; n=20), n (%)	*P* value	
**Inclusion of specific type of information**						
	Dental caries mechanism	20 (48)	12 (55)	8 (40)	.37	
	Any mention of sugar	31 (74)	17 (77)	14 (70)	.73	
	Sugary drinks	20 (48)	11 (50)	9 (45)	.77	
	Snack foods high in sugar and starch	17 (40)	10 (45)	7 (35)	.54	
	Candy	13 (31)	9 (41)	4 (20)	.19	
	Frequency of sugar intake^a^	13 (31)	10 (45)	3 (15)	.047	
	Acidic foods and beverages^a^	11 (26)	9 (41)	2 (10)	.04	
	Sticky foods	10 (24)	6 (27)	4 (20)	.72	
	Vegetables and fruit	15 (36)	8 (36)	7 (35)	.99	
	Brush teeth after eating or brush teeth at least 2 times/day	12 (29)	7 (32)	5 (25)	.74	
	Drink water^b^	12 (29)	9 (41)	3 (15)	.09	
	Protein from high-quality sources	11 (26)	6 (27)	5 (25)	.99	
	Dairy products	9 (21)	5 (23)	4 (20)	.99	
	Sugar-free gum or sugar-free candy or xylitol	6 (14)	5 (23)	1 (5)	.19	
	Whole grains	5 (12)	3 (14)	2 (10)	.99	
	Drink beverages with a straw	2 (5)	0 (0)	2 (10)	.22	
	Mention food guide or food label reading	0 (0)	0 (0)	0 (0)		

^a^*P*<.05 for difference between high–view rate videos and low–view rate videos (Fisher exact test).

^b^*P*<.10 for difference between high–view rate videos and low–view rate videos (Fisher exact test).

In total, 48% (20/42) of the videos contained information on how dental caries are formed. There were no significant differences in the percentage of low–view rate and high–view rate videos that provided this message (*P*=.37).

Overall, 74% (31/42) of the videos contained information about sugar being a cause of dental caries. Of note, guidance on the specific amounts of sugar to consume was not mentioned in any video. Almost half (20/42, 48%) of the videos mentioned that sugary drinks (either in general or specific beverages) were a cause of dental caries. Snack foods high in sugar and starch were mentioned as a risk factor for dental caries in 40% (17/42) of the videos. Candy and sticky foods were mentioned as factors that increase the risk of dental caries in 31% (13/42) and 24% (10/42) of videos, respectively. There were no significant differences in the proportion of low–view rate and high–view rate videos that provided each of the abovementioned messages related to sugary foods and drinks (sugar: *P*=.73; sugary drinks: *P*=.77; snack foods high in sugar and starch: *P*=.54; candy: *P*=.19; and sticky foods: *P*=.72).

Messaging on the frequency of sugar intake was present in 31% (13/42) of the videos. A higher percentage of low–view rate videos contained this message compared with high–view rate videos (10/22, 45% vs 3/20, 15%; *P*=.047).

Acidic foods and beverages being harmful toward oral health were mentioned in 26% (11/42) of videos. This message was more often present in low–view rate videos than in high–view rate videos (9/22, 41% vs 2/20, 10%; *P*=.04).

Compared with harmful foods and behaviors, those that are healthful were mentioned less often. Eating more vegetables and fruit (either in general or specific vegetables or fruits) was the most common healthful behavior mentioned; this message was mentioned in 36% (15/42) of the videos. Eating high-quality protein sources (eg, legumes, pulses, nuts, meat, fish, and seafood) was mentioned in just 26% (11/42) of the videos. In addition, 21% (9/42) of the videos mentioned that dairy products (in general or specific products such as cheese, yogurt, and milk) were beneficial. Whole grains were recommended in 12% (5/42) of the videos. No statistically significant differences were found in the proportion of low–view rate videos versus high–view rate videos that contained each of the healthful food messages listed earlier (vegetables and fruit: *P*=.99; high-quality protein: *P*=.99; dairy products: *P*=.99; and whole grains: *P*=.99).

Drinking water was mentioned as being protective toward dental caries in 29% (12/42) of the videos. Only 2 videos specifically spoke about the consumption of fluoridated water. Drinking water was more often mentioned in low–view rate videos than in high–view rate videos (9/22, 41% vs 3/20, 15% of videos; *P*=.09), but this result was not statistically significant. Brushing teeth at least twice a day or after eating was mentioned in 29% (12/42) of videos. Sugar-free gum or sugar-free candy or xylitol was also discussed in only a few videos (6/42, 14%). Videos rarely recommended drinking beverages with a straw (2/42, 5% of videos). The food guide or food label reading was not discussed in any of the videos. Notably, there were no statistically significant differences in the proportion of low–view rate and high–view rate videos that contained messages for brushing teeth (*P*=.74), sugar-free gum or sugar-free candy or xylitol (*P*=.19), and drinking beverages with a straw (*P*=.22).

## Discussion

### Principal Findings

To our knowledge, this is the first study that has focused on investigating nutrition and dental caries content on YouTube. These results are important because nutrition is strongly related to dental caries risk, dental caries is common, and YouTube is a popular web-based platform for the public to access information. These results provide insights into future directions for YouTube content in this area of public health importance.

Overall, we found that the 42 included videos had a low mean score (4.9, SD 3.4 out of 17 points), indicating that few relevant topics on nutrition and dental caries were covered in the videos. This finding is similar to the findings of other studies that have examined health-related content on YouTube. For example, in a study on oral cancer YouTube videos, Hassona et al [48] found that included videos provided “inadequate descriptions” of oral cancer risk factors. Similarly, in a study on oral hygiene instruction in YouTube videos, Smyth et al [56] found that none of the included videos addressed all topics of interest, and the authors had concerns about the messages presented in some videos. In addition, a recent review article found that the comprehensiveness of YouTube videos on various health topics was low [38]. Similar concerns have also been reported in pediatric oral health education leaflets. Arora et al [57] found that nutrition messaging in these types of leaflets was incomplete. Our results suggest that members of the public accessing YouTube for information on nutrition and dental caries may not get the complete information on this topic needed to fully optimize diets to prevent this issue.

We found that sugar was the most consistent topic mentioned in the included videos (mentioned in 31/42, 74% of videos). No other topic we assessed was mentioned in more than half of the videos. In a content analysis of nutrition information in oral health education leaflets from the United Kingdom, Morgan et al [58] also found that sugar was the most common topic covered and that there was variability in the number of topics covered. We also found that fewer YouTube videos covered foods and beverages to consume to decrease the risk of dental caries (eg, vegetables and fruit). This finding contrasts with the findings of previous studies on oral health leaflets, which showed a high prevalence of messages regarding what foods to consume. For example, Morgan et al [58] found that 73% and 70% of assessed oral health leaflets recommended vegetables and fruit for snacks and drinking only milk and water, respectively. In addition, Arora et al [57] found that 81% of leaflets recommended water and 53% recommended consuming milk. In addition, 44% of the leaflets recommended drinking fluoridated or tap water. Individuals accessing YouTube videos for information on nutrition and dental caries have a high chance of receiving messaging regarding sugar but are less likely to obtain evidence-based messaging about what foods to eat to prevent dental caries. Limited messaging about what foods to eat provides the public with incomplete information, which may affect their ability to make meaningful changes.

As we were conducting our analyses, we noticed that some videos mentioned that concepts surrounding nutrition and dental caries (and specifically sugar) were common knowledge to the public using statements such as “everyone knows,” “most people know,” and “we all know.” These statements contradict studies that have shown that the level of nutritional knowledge related to oral health in different populations may not be ideal [59-61]. When designing future YouTube videos on this topic, it is important to address the amount and frequency of sugar consumption, and it is also important to acknowledge that there are many other foods and eating behaviors that can influence the risk of dental caries, that it is a complex relationship, and that the information may be new to viewers.

Videos created by the government and OHPs had higher mean scores than those produced by health professionals who were not OHPs (including complementary and alternative medicine providers) and individuals with no health professional credentials or unknown credentials. However, these score differences were not statistically significant. Other studies on YouTube video health content generally find that videos produced by health professionals and professional associations are of better quality than those that are not produced by health professionals and professional associations (eg, advertisements) [35,37]. We generally found this to be the case in our study but not always. For example, a couple of videos in our study featuring OHPs had a score of 0. One possible reason could be that the nutrition content in nondietetic health profession programs (including dental programs) is often limited, and there are many barriers toward providing this training; therefore, OHPs may not have in-depth training in this area [62].

Our analysis revealed that there were small nonsignificant negative correlations between various engagement measures (eg, total views, view rate, total likes, like rate, total dislikes, and dislike rate) and video score (out of 17 points; range −0.202 to −0.114). In general, these results align with other content analysis studies on health-related YouTube videos. In a recent review article, Osman et al [38] found that 84% and 74% of the included studies that assessed correlations between engagement and video quality found no correlations or negative correlations for video quality versus number of views and video quality versus number of likes, respectively. However, when we divided our included videos into low–view rate and high–view rate videos, we found that low–view rate videos had a trend toward higher overall score compared with high–view rate videos, but this result was not statistically significant. We also found that low–view rate videos were more likely to have messaging related to the frequency of sugar intake (*P*=.047), acidic foods and beverages (*P*=.04), and water (*P*=.09) compared with high–view rate videos. Messaging regarding the frequency of sugar intake is especially important because the frequency of sugar intake is thought to be possibly more important than the amount of sugar in terms of dental caries risk [63]. In addition, it is expected that individuals will eat sugar; therefore, information on how to best consume this dietary component to prevent dental caries is an important message. Warren et al [37] have previously mentioned that higher engagement with poor-quality videos could suggest that the public may have difficulty determining quality health-related YouTube content. Health professionals have an important role to provide more education to the public about how to select quality videos related to nutrition and dental caries. In addition, oral health, nutrition, and other professionals play important roles in producing evidence-based videos that are engaging and can be easily found by the public. Haslam et al [28] provided a list of strategies that can be used by creators to help make their videos more accessible.

As we watched and scored the videos, we observed that there was some contradictory diet advice related to some evidence-based items included in our 17-item scoring tool, both between videos and within videos, that was worthy of discussion. However, these contradictory messages are not evidence-based and could cause confusion for the public. We will discuss a few examples below, including sugary foods and beverages, whole grains, and milk products.

First, there was some contradictory advice about sugar-rich foods and beverages, where evidence-based guidelines suggest avoidance for dental caries prevention. Juice, which is a sugary beverage, was sometimes recommended or recommended over other sugary drinks. For example, some contradictory advice included recommending calcium-fortified juice, mentioning that unsweetened juice was beneficial for teeth because of vitamin C, and suggesting that juice was not as harmful as other sugary beverages. In addition, dried fruit, a sticky food that is highlighted as a sugary food as part of evidence-based guidelines [1,14,20], was mentioned as healthful in a couple of videos because of the presence of phytochemicals. Although a review article from 2016 has suggested that evidence regarding dried fruit and dental caries is limited [64], these foods are high in sugar. Finally, honey (including manuka honey) was identified as a better sugar choice in a couple of instances. Although a few studies have suggested that honey has antibacterial and antibiofilm properties, clinical evidence of the impact of honey on dental caries is not conclusive, and more studies are needed [65]. In summary, these products are all high in sugar; therefore, caution is needed surrounding these foods regarding dental caries risk.

Second, 2 videos in this data set advised limiting or avoiding whole grains because of concerns surrounding phytic acid causing dental caries, which contradicts evidence-based recommendations to consume whole grains. These videos recommended the consumption of grain products, where phytic acid has been reduced. Phytic acid is an antinutrient found in nuts, seeds, grains, and legumes and is known to bind some trace elements (eg, calcium, iron, and zinc), which can make them unavailable for absorption [66,67]. However, phytic acid should not be a concern when eaten as part of a mixed diet [67], and the benefits of consuming whole grains in Western countries outweigh the potential risks of phytic acid [68]. Currently, there is no strong evidence that phytic acid causes dental caries.

Third, there were a few videos that mentioned consuming dairy products is a risk factor for dental caries (eg, coffee creamers and yogurt owing to the carbohydrate content). Although the main sugar in milk products (lactose) is cariogenic, it is not as cariogenic as other sugars, and milk products contain many other beneficial components for dental caries prevention (eg, casein, calcium, and phosphorus). To date, evidence points toward milk being low cariogenic and possibly anticariogenic [69]. In addition, there was a recommendation to consume raw dairy products. This finding is concerning because raw milk is illegal to sell in many jurisdictions (eg, Canada), and milk pasteurization is often mandatory to avoid severe illnesses [70,71]. Although some cheeses made with raw milk that meet certain criteria can be sold in jurisdictions where pasteurization is mandatory, certain groups (eg, children, older adults, women who are pregnant, and individuals with weakened immune systems) are at risk of harmful effects from consuming these products [71]. Currently, there is no strong evidence suggesting that raw milk is beneficial for preventing dental caries or promoting better oral health.

These contradictory messages may cause confusion to viewers about whether the abovementioned foods or beverages are harmful or healthful regarding oral health and dental caries. These findings are consistent with the findings from the study by Morgan et al [58]. The authors of this UK study found that there were also inconsistencies in the nutrition and oral health information in different leaflets, including confusing information [58]. Arora et al [57] also identified confusing messaging regarding nutrition and oral health in pediatric oral health education leaflets in Australia, including confusing messaging around milk. This observation is important because when the public is exposed to contradictory advice, it has the potential to confuse them. The identification of areas of contradictory information is useful for health professionals looking to develop resources on this topic in the future.

We also found that there were some included videos that mentioned complementary and alternative medicine approaches to optimize oral health that were not included as part of our 17-item scoring tool. Some examples included consumption of probiotic supplements or probiotic-rich foods (mentioned in 4 videos), oil pulling (mentioned in 3 videos, with 1 video stating that this process was unpleasant and not recommended), and various recipes of home remedies for mixtures applied directly to the teeth or mouthwashes with various ingredients such as coconut oil, garlic, mustard oil, turmeric, clove oil, and salt (4 videos). In addition, 8 videos promoted or highlighted the consumption of vitamin K (usually K2) often in conjunction with vitamins A and D. In these videos, foods or supplements for these nutrients were recommended. Vitamin and mineral supplements (eg, vitamin D, calcium, magnesium, and vitamin K2) were also highlighted in 4 videos. Although some of these approaches (eg, probiotics and vitamin D) have generated substantial interest in the research and clinical communities regarding oral health and have shown promise for positive outcomes related to dental caries (eg, probiotics [72-79] and vitamin D [80-82]), for many of these approaches, there is a lack of evidence, and they are not recommended by professional associations (eg, oil pulling is not recommended by the American Dental Association [83], and probiotics are currently not recommended for dental caries prevention by the Canadian Pediatric Society [84]). It is important that health professionals are aware of these types of recommendations being made on the internet and are prepared to answer questions and generate evidence-based content related to these topics to help the public make informed decisions.

### Limitations

A limitation of our study was that although we attempted to imitate search strategies used by the public to capture readily accessed YouTube videos, this might not be a completely accurate representation of the actual approaches used. However, we used Google Keyword Planner to plan searches and selected videos that appeared first in the results. Furthermore, our small sample size might be a limitation, but it is consistent with other studies assessing the health content of YouTube videos [35]. In the future, a study using a larger sample size of videos to evaluate content on this topic may be beneficial. We also excluded videos lasting for >20 minutes. In addition, misinformation was not considered as part of our scoring system. In the future, a study incorporating misinformation into a scoring approach in this topic and including longer videos would be worthwhile.

### Conclusions

Our study found that most YouTube videos regarding nutrition and dental caries feature OHPs, and many videos cover a limited selection of topics. With the high prevalence of dental caries in the general population, the strong link between nutrition and dental caries, and the popularity of YouTube, there is a strong need for quality content containing evidence-based recommendations and information regarding this topic on this platform.

## Abbreviations

OHP: oral health professional
TCPS: Tri-Council Policy Statement
